# Increased Incidence of Endometrial Cancer Following the Women's Health Initiative: An Assessment of Risk Factors

**DOI:** 10.1089/jwh.2018.6956

**Published:** 2019-02-23

**Authors:** Ginger D. Constantine, Grant Kessler, Shelli Graham, Steven R. Goldstein

**Affiliations:** ^1^ EndoRheum Consultants, LLC, Malvern, Pennsylvania.; ^2^ Consultant to EndoRheum Consultants, LLC, Malvern, Pennsylvania.; ^3^ TherapeuticsMD, Boca Raton, Florida.; ^4^ Department of Obstetrics and Gynecology, New York University School of Medicine, New York, New York.

**Keywords:** compounded bioidentical hormone therapy, endometrial cancer, hormone therapy, menopause

## Abstract

***Background:*** The Surveillance, Epidemiology, and End Result (SEER) database shows a variable increase in endometrial cancer incidence over time. The objective of this review was to examine published endometrial cancer rates and potential etiologies.

***Methods:*** Endometrial cancer incidence was obtained from the SEER Program database from 1975 through 2014, and a test for trend in incidence was calculated. Changes in risk factors thought to be associated with endometrial cancer, including age, obesity, diabetes, diet and exercise, reproductive factors, and medications (hormone therapy [HT] including Food and Drug Administration [FDA]-approved and non-FDA–approved [compounded] estrogens and progestogens, tamoxifen, and hormonal contraceptives) were found through PubMed searches. Temporal trends of risk factors were compared with endometrial cancer trends from SEER.

***Results:*** Although endometrial cancer rates were constant from 1992 to 2002 (women 50–74 years of age), they increased 2.5% annually with a 10% increase from 2006 to 2012 (trend test 0.82). Use of approved prescription estrogen–progestogen combination products decreased after the publication of the Women's Health Initiative (WHI) data, whereas other risk factors either remained constant or decreased during the same time; however, compounded bioidentical HT (CBHT) use increased coincident with the endometrial cancer increase.

***Conclusion:*** Endometrial cancer rate increases after the first publication of WHI data in 2002 may be associated with the decreased use of approved estrogen–progestogen therapy, the increase in CBHT use, and the prevalence of obesity and diabetes; potential relationships require further evaluation.

## Introduction

In the United States, uterine cancer is the fourth leading cancer among women, representing 7% of all new cancer cases.^1^ In 2017, ∼61,000 new uterine cancer diagnoses were estimated and nearly 11,000 women were estimated to die from the disease, primarily affecting postmenopausal women (mean age of diagnosis, 62 years).^2^ The greatest percentage of new cases by age from 2010 to 2014 was detected in women around and after menopausal age (16.7% for 45–54 years of age, 34.5% for 55–64 years, and 25.8% for 65–74 years).^2^

The Surveillance, Epidemiology, and End Result (SEER) Program of the National Cancer Institute collects and publishes cancer incidence and survival data from population-based cancer registries covering ∼28% of the U.S. population.^3^ Data from SEER show variable increases in endometrial cancer incidence over time, with sharper incident increases in black women compared with white women.^4^ Another report found the incidence to increase over time in women 50–74 years of age (annual percentage change 2004–2009: 2.8%; 2005–2009: 3.3%; and 2006–2009: 4.2%),^5^ after publication of the Women's Health Initiative (WHI) data.^6^

When the balance of progesterone and estrogen shifts at the time of menopause, with a decrease in progesterone production, even small amounts of circulating estrogens may not be adequately counterbalanced, and can lead to the thickening of the endometrium and potential subsequent endometrial cancer.^7^ Several studies have demonstrated that unopposed estrogen therapy increased the risk for endometrial hyperplasia and cancer, whereas the addition of a progestogen prevents such risk.^8–14^

In addition to the use of menopausal hormone therapy (HT), a number of factors may influence a woman's risk of developing endometrial cancer, including certain medications, obesity, diabetes, reproductive factors, and diet and exercise.^7^ In this study, using publicly available cancer incidence statistics in SEER, we review and quantitatively describe the trend of endometrial cancer incidence in recent years. We also compare the trends in risk factors potentially associated with type I endometrial cancer risk with the incidence rates over time to explore whether these risk factor changes could explain the increase in endometrial cancer observed between 2002 and 2014.

## Methods

Data on endometrial cancer incidence were extracted from January 1975 through December 2014 from the SEER Program database (November 2016 data submission; April 2017 release date).^15^ Informed consent was not applicable since the study was based on a publicly available database. The change in the age-adjusted endometrial cancer incidence from 2006 to 2012 was calculated using a test for trend. Change in the incidence of risk factors for endometrial cancer was then reviewed and compared with the change in SEER incidence of endometrial cancer.

PubMed was searched by two separate individuals to obtain trend data on the following potential risk factors for endometrial cancer: age, obesity (defined as body mass index [BMI] of ≥30 kg/m^2^), medications (HT, including Food and Drug Administration [FDA]-approved and non-FDA–approved [compounded] estrogens and progesterone, tamoxifen, and hormonal contraceptives), diabetes, reproductive factors, diet, and exercise. Search terms included endometrial cancer combined with terms relevant for the mentioned risk factors. The temporal trends of these risk factors potentially associated with endometrial cancer were compared with the endometrial cancer incidence trends.

## Results

### Endometrial cancer incidence in the SEER database

**FIG. 1.**
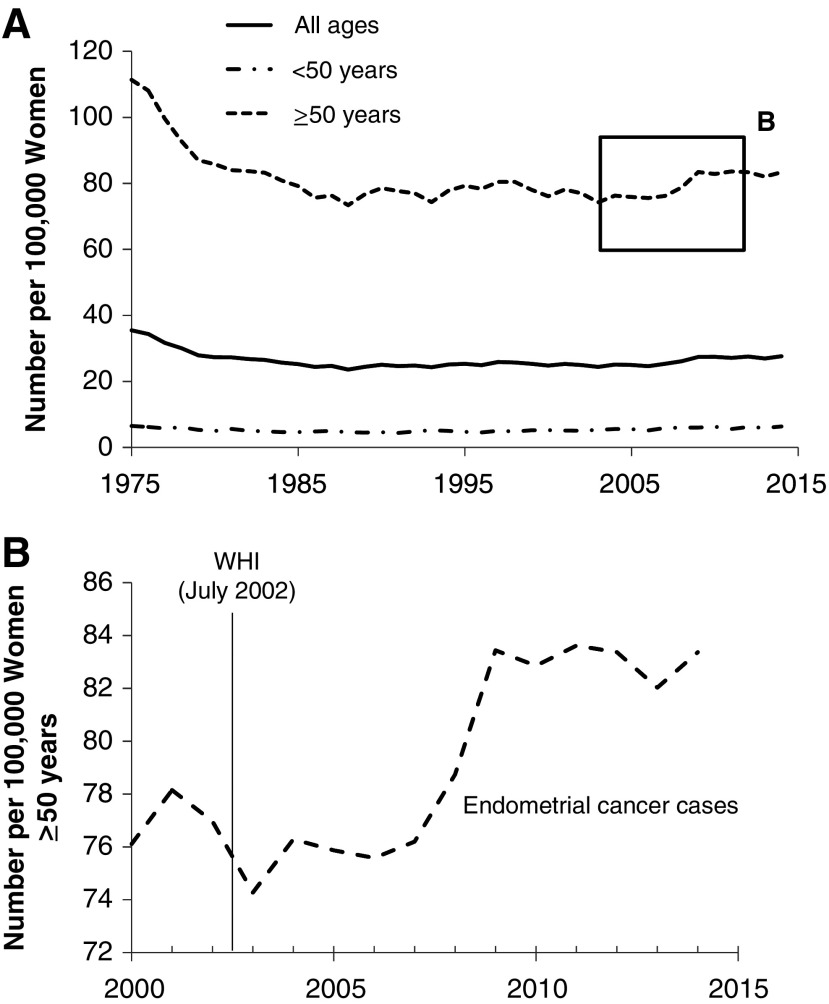
Age-adjusted endometrial cancer incidence per 100,000 from SEER for **(A)** all women between 1975 and 2014 and **(B)** in women ≥50 years for 2000–2014.^4^ SEER, Surveillance, Epidemiology, and End Result.

Data from SEER showed that endometrial cancer rates declined between 1975 and 1992, and then remained relatively constant up to 2002 in women 50 years of age or older (Fig. 1A).^4^ Extracting data from the SEER database, we calculated that the age-adjusted incidence rate per 100,000 people increased 2.5% annually with a 10% increase from 2006 to 2012, post-WHI trial (test for trend 0.82, *R*^2^ = 0.65; Fig. 1B).

### Menopausal HT

#### Decrease in FDA-approved estrogen and progesterone therapies

Increased risk for endometrial cancer was unequivocally shown in earlier studies of unopposed oral estrogen.^16–19^ A rising incidence of endometrial cancer was also reported after estrogen therapy first became commonly used to treat menopausal symptoms and as use increased in the 1970s.^20^ Early data also showed that addition of a progestogen prevented the increased risk of endometrial cancer with unopposed estrogens.^18,19^ Later studies confirmed this association,^11,21^ including data from the WHI showing a decrease in endometrial cancer with continuous estrogen plus progestogen compared with placebo.^22^ Currently, women with a uterus taking systemic estrogens (oral/transdermal) are to be prescribed a progestogen to prevent endometrial hyperplasia, and the potential for subsequent endometrial cancer.^23^

**FIG. 2.**
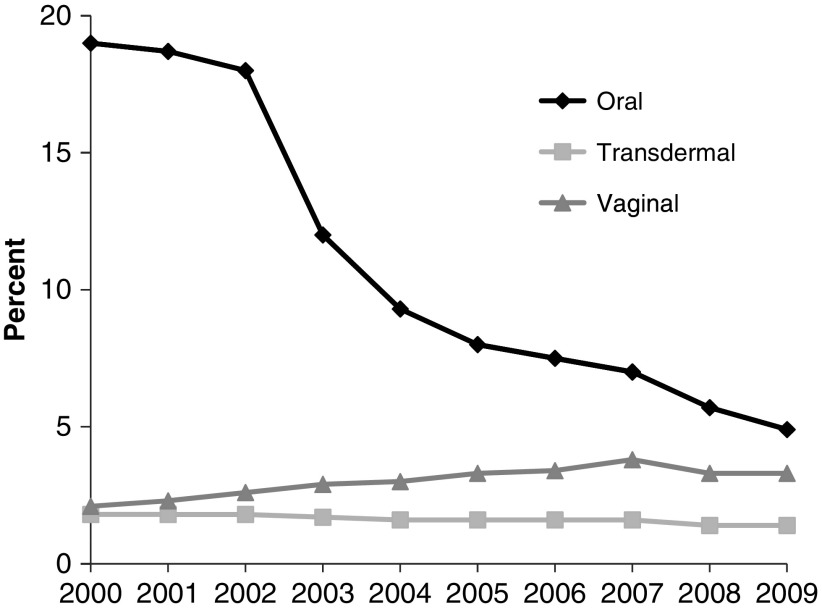
Prevalence of women using hormone therapy by formulation between 2000 and 2009.^25^

After the first publication of the WHI findings,^6^ prescriptions of FDA-approved HT significantly decreased among postmenopausal women.^24,25^ Before the WHI publication, annual HT use had increased from 58 million prescriptions in 1995 to 90 million prescriptions in 1999 and remained stable through June 2002.^26^ Within 1 year of the WHI publication, use of prescribed HT sharply declined to the levels of 1995,^26^ and continued to decline until 2009 (Fig. 2).^25^ The authors of one study suggest that the increased incidence of endometrial cancer observed after 2002 may possibly be linked to the decreased use of menopausal HT with a progestogen.^5^

#### Increase in FDA-approved vaginal estrogens

Medical societies have suggested that the use of a progestogen is not always required with vaginal estrogens as local vaginal estrogen use has not been associated with increased risk of endometrial cancer,^27–30^ and does not result in endometrial hyperplasia rates as reported for systemic unopposed estrogens. However, large long-term studies of vaginal estrogens are lacking. Although use of vaginal estrogens almost doubled from 2000 to 2009 (Fig. 2) and later decreased from 2011 through 2015,^25,31^ clinical trials and observational studies of vaginal estrogen use have not found an association with endometrial cancer.^27–30,32^ Therefore, it is unlikely that vaginal estrogens contribute to the increase in endometrial cancer observed between 2002 and 2009.

#### Increase in compounded bioidentical HT

Compounded bioidentical HT (CBHT), which is not FDA-approved and is inadequately regulated by the FDA, is another form of menopausal HT. Clinicians have noticed a precipitous increase in the use of CBHT since the publication of the WHI findings, although quantifying that increase has been challenging since CBHT use is not systematically tracked and available material on the magnitude of its use is limited.^33–35^ However, some recent publications have estimated CBHT use and have reported an increase in its use. Two smaller surveys (*n* = 160 responders in each) by the same authors showed an increase of 23% in any compounding prescription from 2005 to 2006.^36,37^ In a 2012–2013 analysis of a large national prescription claims database (>22 million members), a 27% increase in the use of any compounded medication and a 34% increase in claims for the same were observed from 2012 to 2013, and CBHT was the most commonly compounded therapy among women aged 40–69 years, with this gender and age group using compounded therapies most frequently.^38^ A 2014 survey of compounding and independent community pharmacists reported that their compounding business had grown or stayed the same in the past 2 years, and most expected continued growth, anticipating 5%–25% growth of CBHT use over the next 2 years.^39^ A study, based on two surveys conducted in 2013 and 2014, estimated that 1–2.5 million women older than 40 years of age may be using CBHT a year (an estimated 21–39 million prescriptions).^40^ Finally, a 2015 North American Menopause Society survey reported that 35% of U.S. women currently using HT (and 41% of ever users aged 40–49 years) are using CBHT.^41^

The incidence of endometrial hyperplasia with CBHT has not been rigorously studied as with FDA-approved HTs. The levels of progesterone in CBHT, as in any HT, must be high enough to counter any endometrial stimulation by estrogens. Some cases of endometrial cancer and endometrial hyperplasia,^41–44^ as well as higher rates of vaginal bleeding,^41^ have been reported among healthy CBHT users.

The increase in CBHT use is consistent with the increased incidence of endometrial cancer after the WHI in 2002, implicating a potential role for CBHT in this endometrial cancer increase.

### Obesity

**FIG. 3.**
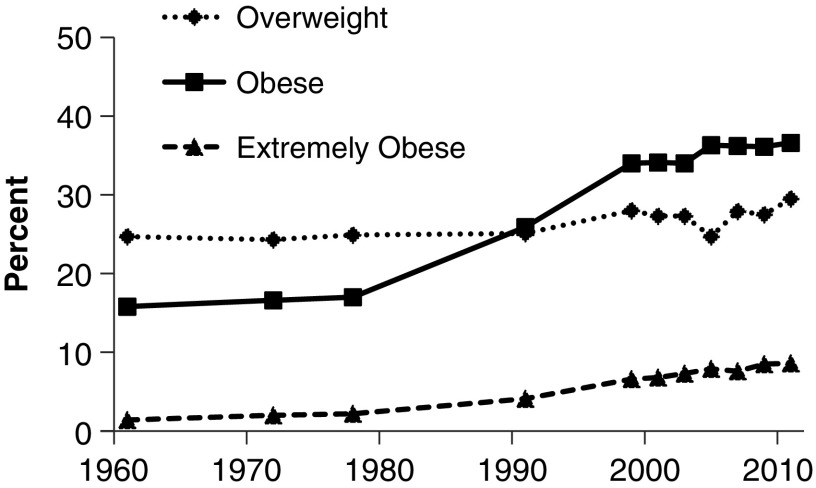
Age-adjusted prevalence of overweight (BMI 25 to <30 kg/m^2^), obese (BMI ≥30 kg/m^2^), or extremely obese (BMI ≥40 kg/m^2^) women 20–74 years of age between 1960 and 2012.^50^ BMI, body mass index.

Obesity may account for up to 40% of the observed endometrial cancer incidence, with obese women having a twofold to fivefold increased risk of developing endometrial cancer compared with normal weight women.^45–47^ In general, obesity is associated with higher levels of circulating estrogens in postmenopausal women,^48,49^ likely accounting for their increased risk of endometrial cancer. In the United States, the age-adjusted prevalence of obesity (body mass index [BMI] ≥30 kg/m^2^) in women remained stable from 1960 and 1980, which was followed by a sharp increase between 1980 and 1999 (Fig. 3).^50^ From 1999 to 2014, the prevalence of obesity increased among all women, including women who were 45–64 and ≥65 years^51^; however, this increase was minimal and of smaller magnitude compared with the increase in endometrial cancer. A role for obesity in the increased incidence of endometrial cancer after WHI is not likely (or a very small contributor), given its much smaller change in prevalence over the same time period.

### Diabetes

Diabetes has also been associated with a significant increased risk of endometrial cancer.^52^ Data based on the National Health Interview Survey (NHIS) showed that the prevalence of diabetes in women increased between 1990 and 2008, before leveling off with no significant change between 2008 and 2012.^53^ U.S. adults aged ≥65 years were twice as likely to have diabetes (26.9% vs. 13.7%) than younger adults (45–64 years) from 2005 to 2008.^54^ Although these data suggest that the increase in diabetes observed may have contributed to the increase in endometrial cancer incidence, it would not explain the abrupt increase observed between 2002 and 2009 and maintained through 2014.

### Reproductive factors

Certain reproductive factors may also influence the incidence of endometrial cancer by affecting the relative estrogen/progesterone balance.^55^ Women with an increased lifetime exposure to estrogens, including women with an early age at menarche, later age at menopause, lower parity, and no history of oral contraceptive use, have been linked to a higher incidence of endometrial cancer.^45,55–57^

Overall, all of these reproductive factors have remained fairly constant during the time of observed endometrial cancer increase. The age of menarche has remained relatively the same over the past 40 years in the Unites States, although the age of thelarche seems to be occurring earlier.^58,59^ Data from the 2012 American Community Survey and Birth Record Data from Vital Statistics show that although the number of children born per woman decreased between 1976 and 1992, the rate remained fairly uniform between 1992 and 2012.^60^

Use of oral contraceptives reported by the National Survey of Family Growth also shows fairly constant use between 1982 and 2008, with rates of 15.6% in 1982, 17.3% in 1995, 18.9% in 2002, and 17.3% between 2006 and 2008.^61^ A later analysis of this database showed no significant change in the prevalence of oral contraceptive use from 2008 to 2014.^62^ Despite this stable prevalence, a change in oral contraceptive formulations resulting in lower progesterone relative to estrogen could potentially increase endometrial cancer risk. However, the dominant trend in formulation changes for oral contraceptives has been a decrease in estrogen rather than a decrease in progesterone. Furthermore, oral contraceptives are well known to reduce the risk of endometrial cancer, with risk reduction that increases with duration of use and persists up to 30 years after their discontinuation.^63–65^

Intrauterine devices (IUDs) were shown to have a protective effect against endometrial cancer, which is increased with duration of use, and sustained up to 5 years after cessation of use.^66,67^ National Survey of Family Growth data have shown a high increase in the popularity of long-acting reversible contraceptives (LARCs; IUDs and implants) in women aged 15–44 years between 1982 and 2013.^68^ Usage of LARCs was constant between 1998 and 2002 (1.5%); current LARC use then doubled in 2006–2010 (3.8%) and then nearly doubled for 2011–2013 (7.2%).^68^ No studies have yet reported whether newer types of IUDs that release progesterone have any effect on endometrial cancer risk; however, these IUDs are sometimes used to treat precancers and early endometrial cancers.^69^

Based on the described trends, reproductive factors do not appear to be major contributors to the increase of endometrial cancer incidence after WHI.

### Cancer and cancer-related treatments

Although rare, there are certain types of cancers that have estrogen-secreting capabilities and may contribute to endometrial tissue stimulation. Granulosa cell tumors (GCTs) of the ovary represent ∼5% of all ovarian malignancies, and adult GCTs occur primarily in perimenopausal or early postmenopausal women.^70^ Evidence suggests that GCTs secrete increased levels of estradiol, and elevated estradiol serum levels may be responsible for disease progression and clinical manifestations of other diseases.^70^ Nevertheless, in addition to the low occurrence of GCTs of the ovary, there has been an overall decrease in invasive ovarian cancer in women ≥65 years from 52.5% in 1998 to 41.6% in 2014,^71^ suggesting that this risk factor would not contribute to the observed increase in endometrial cancer incidence.

Cancer-related treatments, such as the selective estrogen receptor modulator tamoxifen, may also affect endometrial cancer risk. Although effective in reducing breast cancer incidence by acting as an antiestrogen in breast tissue, evidence suggests that tamoxifen acts as an estrogen in the uterus and increases endometrial cancers.^7,72^ Despite the increased risk of tamoxifen use with endometrial cancer, tamoxifen use has decreased since 2000 and thus is unlikely to be a significant risk factor.^73^

### Exercise

Epidemiological evidence has shown that physical activity can lower the risk of endometrial cancer by 20%–40% compared with physical inactivity.^74–76^ To date, the prevalence of leisure-time physical inactivity has gradually declined over the past three decades in most U.S. states; however, rates of physical inactivity remain substantially high.^77^ Based on these data, the small changes observed in exercise among U.S. adults may not have significantly affected overall endometrial cancer incidence after WHI.

## Discussion

Owing to the significant increase in endometrial cancer incidence observed post-WHI, we investigated the potential contribution of each risk factor to this increased incidence. As reviewed here and as suggested by others, changes in the prevalence of known endometrial cancer risk factors seemed unlikely to account for the increase in endometrial cancer incidence.^5^ Our data review demonstrates a decrease in the use of FDA-approved estrogen plus progestogen HT (known to protect the endometrium), which has been shown to be associated with an increase in endometrial cancer risk. Thus, this decrease may be a contributing factor to the increased endometrial cancer incidence after the WHI. Similarly, the increase in CBHT use may also increase the incidence of endometrial cancer, with obesity and diabetes contributing to the increase in incidence but likely to a much lesser degree.

Unlike manufactured FDA-approved HT,^40,41^ CBHT is not approved nor regulated by the FDA and, therefore, does not carry the safety warnings mandated for all HTs by the FDA after the WHI.^78^ Since it also lacks standard guidelines set forth by the FDA, under- and overdosage of CBHT are possible due to its inconsistent bioavailability and bioactivity.^35^ The variability in purity and quality of progesterone products was examined and two out of five progesterone samples (40%) tested failed repeat potency testing and one sample (20%) failed uniformity testing (consistent dosages), leading to underdosing with progesterone.^79^ Another sampling of compounded progesterone vaginal suppositories from 10 randomly chosen pharmacies found that half (5/10) of the pharmacies provided product that was subpotent or superpotent (outside of the 90%–110% acceptable range).^80^ Similarly, CBHT prescriptions from 12 sources had superpotent estradiol or estrone (up to >250%) and subpotent progesterone (60%–80%) in a small investigation by one physician.^81^ Although we cannot be certain that these small studies of CBHT potency are representative of most CBHT being used, there is still cause for concern given the considerable risks: underdosing of progesterone and/or overdosing of estrogens could lead to an increased risk of endometrial hyperplasia and cancer, overdosing of estrogens to an increased risk of venous thromboembolism, and underdosing of estrogens to an increased risk of osteoporosis.^35^

In addition, numerous claims regarding CBHT in comparison with FDA-approved HT products have been noted; however, none of them have been supported by scientific evidence, and clinicians should carefully consider each claim with caution before prescribing CBHT.^35,82,83^ The lack of reliable placebo- or comparator-controlled studies evaluating CBHT makes it difficult to accurately assess CBHT efficacy or safety. As stated by the American College of Obstetricians and Gynecologists, the North American Menopause Society, the Endocrine Society, and the American Society for Reproductive Medicine, CBHT poses additional risk to its users due to its variable purity, potency, and lack of efficacy and safety data^23,35,78,84^ and, therefore, should be avoided unless medically indicated (dosing or formulation not available or allergy to FDA-approved products).^23^ Patients and physicians should equally educate themselves on the risks and benefits of CBHT, and physicians should be cautious in prescribing CBHT due to the perceived risk of increasing endometrial cancer risk. Women currently taking CBHT should also receive regular endometrial surveillance as a safety precaution.

In conclusion, an increase in endometrial cancer incidence may be associated with a number of risk factors, including increased CBHT use, obesity, and diabetes, as well as decreased use of approved estrogen–progestogen HT. We believe that CBHT use to be the most likely significant risk factor for the observed increase in endometrial cancer post-WHI. Additional studies are needed to fully assess unapproved CBHT as a risk factor for endometrial cancer and to further explore the significant health implications of CBHT use in postmenopausal women.
